# Characterization of a bacterial tyrosine kinase in *Porphyromonas gingivalis* involved in polymicrobial synergy

**DOI:** 10.1002/mbo3.177

**Published:** 2014-05-09

**Authors:** Christopher J Wright, Peng Xue, Takanori Hirano, Chengcheng Liu, Sarah E Whitmore, Murray Hackett, Richard J Lamont

**Affiliations:** 1 Oral Health and Systemic Disease, University of LouisvilleLouisville, Kentucky, 40202; 2 Key Laboratory of Molecular and Cellular Immunology, Henan UniversityKaifeng, 475004, China; 3 University of Florida, Department of Oral BiologyGainesville, Florida, 32610; 4 State Key Laboratory of Oral Diseases, West China Hospital of Stomatology, Sichuan UniversityChengdu, 610041, China; 5 Department of Chemical Engineering and Center for Microbial Proteomics, University of WashingtonSeattle, Washington, 98195

**Keywords:** Community, periodontitis, polymicrobial synergy, *Porphyromonas gingivalis*, tyrosine kinase

## Abstract

Interspecies communication between *Porphyromonas gingivalis* and *Streptococcus gordonii* underlies the development of synergistic dual species communities. Contact with *S. gordonii* initiates signal transduction within *P. gingivalis* that is based on protein tyrosine (de)phosphorylation. In this study, we characterize a bacterial tyrosine (BY) kinase (designated Ptk1) of *P. gingivalis* and demonstrate its involvement in interspecies signaling. Ptk1 can utilize ATP for autophosphorylation and is dephosphorylated by the *P. gingivalis* tyrosine phosphatase, Ltp1. Community development with *S. gordonii* is severely abrogated in a *ptk1* mutant of *P. gingivalis*, indicating that tyrosine kinase activity is required for maximal polymicrobial synergy. Ptk1 controls the levels of the transcriptional regulator CdhR and the fimbrial adhesin Mfa1 which mediates binding to *S. gordonii*. The *ptk1* gene is in an operon with two genes involved in exopolysaccharide synthesis, and similar to other BY kinases, Ptk1 is necessary for exopolysaccharide production in *P. gingivalis*. Ptk1 can phosphorylate the capsule related proteins PGN_0224, a UDP-acetyl-mannosamine dehydrogenase, and PGN_0613, a UDP-glucose dehydrogenase, in *P. gingivalis*. Knockout of *ptk1* in an encapsulated strain of *P. gingivalis* resulted in loss of capsule production. Collectively these results demonstrate that the *P. gingivalis* Ptk1 BY kinase regulates interspecies communication and controls heterotypic community development with *S. gordonii* through adjusting the levels of the Mfa1 adhesin and exopolysaccharide.

## Introduction

Infections that originate at mucosal membranes are often polymicrobial, and bacterial constituents of heterotypic communities exhibit synergistic interspecies interactions. Periodontitis, one of the most common infectious diseases of humans, is an exemplar of a mixed microbial infection. The initiation and progression of periodontitis depends on the action of a dysbiotic microbial community which elicits a malfunctioning immune response (Hajishengallis and Lamont 2012, 2014). *Porphyromonas gingivalis*, a Gram-negative anaerobe, is a keystone pathogen in periodontal disease, capable of elevating the pathogenicity of the entire microbial community (Hajishengallis et al. 2011). The virulence of *P. gingivalis* is enhanced by communication with accessory pathogens such as *Streptococcus gordonii* (Daep et al. 2011; Whitmore and Lamont 2011). *P. gingivalis* and *S. gordonii* accumulate into dual species communities following interspecies co-adhesion mediated through the FimA and Mfa1 component fimbrial adhesins of *P. gingivalis* which bind the GAPDH and SspA/B streptococcal surface proteins, respectively (Maeda et al. 2004; Park et al. 2005; Kuboniwa and Lamont 2010). Subsequently, a signal transduction cascade is activated within *P. gingivalis* that ultimately downregulates expression of Mfa1 and LuxS the enzyme which produces the AI-2 family of signaling molecules (Simionato et al. 2006; Maeda et al. 2008; Chawla et al. 2010). Reduction in the adhesive and signaling capacity of *P. gingivalis* leads to constraint of further heterotypic community development. However, the proteolytic activity of *P. gingivalis* is increased, and communities of *P. gingivalis* and *S. gordonii* are more pathogenic in animal models of periodontal diseases compared to either species alone (Daep et al. 2011; Whitmore and Lamont 2011).

The components of the signaling pathway in *P. gingivalis* that are activated by *S. gordonii* and control community development and polymicrobial synergy are under investigation. Engagement of Mfa1 increases expression of a tyrosine phosphatase (Ltp1), and dephosphorylation reactions converge on the transcriptional regulator CdhR (Maeda et al. 2008; Chawla et al. 2010). CdhR represses expression of both *mfa1* and *luxS,* and mutations in either *ltp1* or *cdhR* result in higher levels of accumulation with *S. gordonii*. The involvement of a tyrosine phosphatase led us to hypothesize that a cognate tyrosine kinase would be present in *P. gingivalis* and contribute to the regulatory circuitry.

Initially thought of as an exclusively eukaryotic trait (Cozzone et al. 2004), phosphorylation on tyrosine has emerged as a widespread phenomenon in bacteria (Cozzone 2005; Grangeasse et al. 2007, 2012). Bacterial tyrosine (BY) kinases are structurally distinct from eukaryotic tyrosine kinases, but are well conserved among bacterial species (Cozzone 2009; Grangeasse et al. 2012). In Gram-negative organisms, a single gene encodes a membrane-associated protein with periplasmic domains as well as the functional Walker motifs necessary for phosphotransfer, both autophosphorylation and phosphorylation of endogenous substrates. The best documented role for BY kinases is in the synthesis of extracellular polysaccharide where they function in polymerization and transport (Whitfield 2006). However, phosphoproteomic studies have revealed a wide range of potential substrates for BY kinases and they are increasingly recognized as regulators of a variety of bacterial functions (Grangeasse et al. 2010, 2012; Whitmore and Lamont 2012; Hansen et al. 2013).

In this study, we identify a tyrosine kinase in *P. gingivalis* and characterize its role in extracellular polysaccharide production and heterotypic community development with *S. gordonii*.

## Experimental Procedures

### Bacterial strains and growth conditions

*Porphyromonas gingivalis* strains ATCC 33277 and W83 were cultured anaerobically at 37°C in Trypticase soy broth (TSB) supplemented with 1 mg/mL yeast extract, 5 *μ*g/mL hemin, and 1 *μ*g/mL menadione. For solid medium, TSB broth was supplemented with 5% sheep blood and 1.5% agar. When required, erythromycin (10 *μ*g/mL) and tetracycline (1 *μ*g/mL) were added to solid or liquid media. *S. gordonii* was cultured in Brain Heart infusion (BHI) broth containing 0.5% yeast extract. *E. coli* strains were grown aerobically with shaking at 37°C in Luria-Bertani broth containing kanamycin (50 *μ*g/mL) or ampicillin (100 *μ*g/mL) when required.

### Mutant construction

The PCR fusion technique (Simionato et al. 2006) was employed to generate allelic exchange mutants of *ptk1* in ATCC 33277 and W83 using primers listed in Table S1. Upstream and downstream fragments of approximately 800 bp flanking *ptk1* (PGN_1524 in 33277; PG0436 in W83) were amplified by PCR from *P. gingivalis* genomic DNA and fused to the *ermF* gene. The resulting construct was introduced into the appropriate *P. gingivalis* strain by electroporation, and transformants selected for resistance to erythromycin. Insertion of the construct was confirmed by PCR. To generate Δ*ptk1 +* p*ptk1*, a complemented strain of Δ*ptk1* in 33277, the promoter region upstream of PGN_1523 and the coding region of *ptk1* were amplified and fused together, and the construct was cloned into pT-COW (Gardner et al. 1996). The resulting plasmid pT1524 was electroporated into Δ*ptk1* and selected on TSB agar containing erythromycin and tetracycline. Plasmids were sequenced to confirm accuracy. Parent and mutant strains showed no difference in growth rate (Fig. S1).

### Expression and purification of recombinant proteins

The C-terminal region (aa 541-821) of *ptk1* was amplified by PCR using primers 1524pet200F and 1524pet200R (Table S1) yielding a product of approximately 850 bp. This was cloned into the expression vector pET200 (Invitrogen, Carlsbad, CA) to create pET200-F*ptk1* and transformed into *E. coli* TOP10 cells (Invitrogen). Successful transformants were analyzed by colony PCR and constructs containing the correct insert were sequenced to ensure accuracy. For protein purification, pET200-F*ptk1* was transformed into the expression strain BL21 Star (Invitrogen). His-tag protein was purified with the Membrane Protein Purification Kit (GE Healthcare, Waukesha, WI) using detergent n-Dodecylphosphocholine for solubilization. Purity of FPtk1 was assessed using sodium dodecyl sulfate polyacrylamide gel electrophoresis (SDS-PAGE) and Coomassie staining.

Purification of Ltp1 was carried out using MagneHIS particles (Promega, Madison, WI) following the manufacturer's instructions, as a modification of the method described previously (Maeda et al. 2008). To generate recombinant Ltp1:C^10^S, the coding region for the catalytically inactive Ltp1:C^10^S was amplified by PCR from pTCOW-Ltp1:C^10^S (Maeda et al. 2008) using primers listed in Table S1, and cloned into the expression vector pET200. Following sequencing, the pET200 vector containing Ltp1:C^10^S was transformed into BL21 Star as described above. Soluble protein was obtained using MagneHIS particles. The purity of the resulting protein was determined by SDS-PAGE and Coomassie staining. Recombinant PGN_0224, PGN_0613, and PGN_0261 were obtained by the amplification of the entire coding region from a *P. gingivalis* 33277 genomic template using primers listed in Table S1 and cloning into pET200. Soluble protein was obtained as described above.

### Quantitative gene expression

Total RNA was extracted with Trizol (Invitrogen) from *P. gingivalis* cells reacted with *S. gordonii,* or held in phosphate buffered saline (PBS), anaerobically for 1 h. RNA was treated with DNase I and 20 ng of RNA template was converted to cDNA using the high capacity cDNA reverse transcription kit (Applied Biosystems, Carlsbad, CA). Quantitative RT-PCR (qPCR) was performed on a StepOne Plus Real-Time PCR system (Applied Biosytems) using Power SYBR green PCR master mix, 250 nmol/L of primers (Table S1) and the following conditions: 1 cycle of 95°C for 30 sec, 40 cycles of 95°C for 3 sec and 60°C for 30 sec. Melting curves were acquired on the SYBR green channel from 60°C to 95°C with 0.3°C increments. Relative quantities of mRNA expression were normalized to 16S rRNA by the ΔΔCt method. Conditions with no RT were included as controls in all experiments.

### 5′ RACE and RT-PCR

To determine the operon structure of the *ptk1* locus, reverse transcriptase (RT) PCR was employed. Total RNA was extracted using Trizol, treated with Turbo-DNA free (Invitrogen) and converted to cDNA as described above. Primers described in Table S1 were used in PCR reactions with *P. gingivalis* genomic DNA, cDNA, and a no reverse transcriptase control. The transcriptional start site (tss) was mapped using 5′ rapid amplification of cDNA ends (RACE) as described previously (Hirano et al. 2013). Briefly, RNA was further purified using an RNeasy kit with on column DNase I treatment (Qiagen, Valencia, CA). The FirstChoice RLM-RACE kit (Ambion, Carlsbad, CA) was employed according to the manufacturer's instructions. The resulting fragments generated by PCR were TA cloned into vector pCR 2.1 (Invitrogen) before being sequenced for 5′ detection.

### *P. gingivalis*–*S. gordonii* communities

Heterotypic communities of *P. gingivalis* and *S. gordonii* were generated and analyzed as described previously (Kuboniwa et al. 2006). Approximately 2 × 10^8^ *S. gordonii* cells stained with hexidium iodide (15 *μ*g/mL, Invitrogen) were incubated on glass coverslips for 16 h anaerobically at 37°C. *P. gingivalis* cells were labeled with 5-(and-6)-carboxyfluorescein, succinimidyl ester (FITC, 4 *μ*g/mL, Invitrogen) and 2 × 10^7^ cells were reacted with *S. gordonii* for 18 h in prereduced PBS anaerobically at 37°C with rocking. The resulting community was washed with PBS before analysis by confocal scanning laser microscopy (CSLM) on an Olympus FV500 confocal microscope. XYZ stacks were digitally reconstructed using the Volocity analysis program (Perkin Elmer, Waltham, MA). Quantitation of the volume of *P. gingivalis* fluorescence was obtained using the Find Objects algorithm in the Volocity program. This process analyzed all *P. gingivalis* fluorescence in the 3D digitally recreated confocal images. To estimate microcolony formation, the Find Objects process was used with a threshold for 3D objects greater than 30 *μ*m^3^.

### Transmission electron microscopy

Mid-exponential phase cells of W83 and Δ*ptk1* were centrifuged for 10 min (3000*g*) before washing in PBS. Cells were fixed for 2 h at room temperature in 0.1 mol/L phosphate buffer (pH 7) containing 3.6% (vol/vol) glutaraldehyde, ruthenium red (0.075%), and lysine (55 mmol/L). Following fixation, cells were briefly washed in PBS before secondary staining for 1 h at room temperature in 0.1 mol/L phosphate buffer (pH 7) containing 2% osmium tetroxide. Grids were visualized on a Phillips CM10 electron microscope.

### Extracellular polysaccharide detection

Exopolysaccharide production was determined with fluorescent lectins as described previously (Maeda et al. 2008). *P. gingivalis* cells were labeled with Syto-17 (Invitrogen) and deposited on glass coverslips. Polysaccharide was labeled with concanavalin A-FITC and wheat germ agglutinin-FITC (100 *μ*g/mL) for 30 min at room temperature. After washing, images were collected by confocal microscopy and analyzed as described above.

### Quantitative kinase assay

Kinase activity was measured using the Beacon tyrosine kinase assay (Molecular Probes, Carlsbad, CA). Ptk1:541-821 was incubated with 0.5 mmol/L ATP and a poly Glu:Tyr (4:1 ratio) substrate (200 *μ*g/mL) for 15 min before measuring the fluorescence intensity using a Victor X1 plate reader.

### Dephosphorylation assay

Ltp1 or Ltp1:C^10^S (5 *μ*g) were preincubated with 5 *μ*g of Ptk1:541-821 for 1 h, separated by 10% SDS-PAGE and transferred to a nitrocellulose membrane. Membranes were blocked for 16 h at 4°C in Tris buffered saline containing 0.1% Tween-20 (TBST) and 10% bovine serum albumin. Following blocking, membranes were washed in TBST and probed with a 1:1000 dilution of phosphotyrosine antibodies (Clone PY20; Sigma-Aldrich, St Louis, MO) for 2 h. Membranes were washed three times with TBST before addition of a horseradish peroxidase-conjugated secondary antibody (1:1000) and incubation for 2 h at room temperature. Immunoreactive bands were detected on membranes using Enhanced chemiluminescence (ECL) reagent and a ChemiDoc XRS imaging system (BioRad, Hercules, CA).

### Auto- and substrate phosphorylation

For autophosphorylation, Ptk1:541-821 (5 *μ*g) was treated with 20 units of calf intestinal alkaline phosphatase (New England Biosciences, Ipswich, MA) in manufacturer supplied buffer 3 for 1 h at 37°C. ATP (0.1 mmol/L) and phosphatase inhibitor cocktail 2 (Sigma-Aldrich) were added for an additional 15 min before reactions were stopped with an equal volume of 2× SDS-PAGE sample buffer and boiling for 10 min. Samples were separated by SDS-PAGE followed by Western blotting with phosphotyrosine antibodies and ECL detection as described above.

For the substrate phosphorylation assay, 5 *μ*g of Ptk1:541-821 were incubated with 10 *μ*g of recombinant substrate. Five millimolar ATP and 0.5 *μ*L of phosphatase inhibitor cocktail 2 were added and the reaction mixture was incubated for 30 min at 37°C. The reaction was stopped with the addition of 2× SDS sample buffer and boiling 10 min. Samples were analyzed by Western immunoblotting as described above.

### Statistics

Experiments were carried out in triplicate and Prism 6 (Graphpad, La Jolla, CA, USA) software was used to analyze data displayed as mean ± standard deviation (SD). Multiple data sets were analyzed by analysis of variance followed by Tukey post test. Duplicate data sets were analyzed by Student's *t*-test. Data presented are representative of at least three independent experiments.

## Results

### PGN_1524 is a BY kinase in *P. gingivalis*

**Figure 1:**
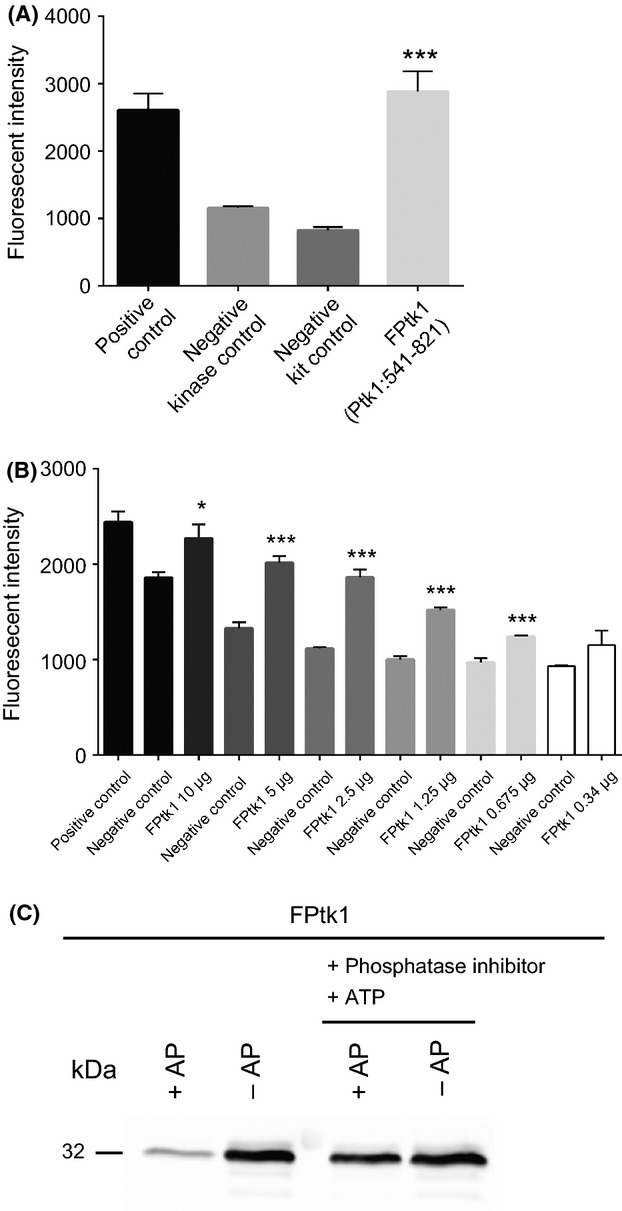
Ptk1 demonstrates ATPase activity and autophosphorylation. (A) Tyrosine kinase assay (Beacon) with the 541-821 C-terminal amino acid fragment of Ptk1 (FPtk1, 5 *μ*g). Positive control and negative kit control were supplied in the assay kit. Negative kinase control is buffer only. Error bars are SD, *n* = 3. ***P < 0.001 compared to kinase control. (B) Concentration-dependent kinase activity of FPtk1. Negative control is kinase buffer. *P < 0.05; ***P < 0.001 compared to negative control. (C) Autophosphorylation of FPtk1. FPtk1 was dephosphorylated with alkaline phosphatase (AP) followed by treatment with phosphatase inhibitor and ATP or left untreated as a control. Phosphorylated FPtk1 was visualized by Western blotting with phosphotyrosine antibodies.

In silico interrogation of the *P. gingivalis* 33277 genome using BLASTP, Pfam, and Lalign revealed that PGN_1524, currently annotated as a hypothetical protein (http://img.jgi.doe.gov), possesses features typical of a proteobacterial BY kinase (Grangeasse et al. 2012), namely a transmembrane domain, Walker A, A' and B motifs (beginning at aa residue 613), and a C-terminal tyrosine-rich cluster. Comparison of the amino acid sequence of PGN_1524 with the Wzc tyrosine kinase of *E. coli* (Grangeasse et al. 2012) showed 56.7% similarity and 23.2% identity. To confirm tyrosine kinase activity we first attempted to express and purify recombinant full length protein. However, full length Ptk1 appeared to be toxic in an *E. coli* host and this approach was unsuccessful. As the cytoplasmic domain of other BY kinases has been shown to demonstrate functional activity (Grangeasse et al. 2002), we adopted this strategy for PGN_1524. A cytoplasmic domain comprising amino acids residues 541-821 (Ptk1:541-821) was expressed in *E. coli* cells with minimal toxicity. When this fragment was tested in a tyrosine kinase assay, significant kinase activity was observed (Fig. 1A), and activity was dose-dependent (Fig. 1B). Thus, we designated PGN_1524 as Ptk1 (*Porphyromonas gingivalis* tyrosine kinase 1).

A feature of BY kinases is the ability to autophosphorylate (Obadia et al. 2007), and hence we next tested the autophosphorylation capacity of the *P. gingivalis* kinase. The Ptk1:541-821 C-terminal region was significantly phosphorylated when purified from *E. coli*, and so we dephosphorylated Ptk1:541-821 with alkaline phosphatase. After inhibiting the phosphatase, the addition of exogenous ATP restored the phosphorylated state of Ptk1:541-821 (Fig. 1C). This result demonstrates that Ptk1 is capable of utilizing ATP for autophosphorylation and the activity resides in the cytoplasmic domain, concordant with BY kinases in other Gram-negative bacteria (Cozzone et al. 2004; Grangeasse et al. 2012).

### Transcriptional organization of the *ptk1* locus

**Figure 2:**
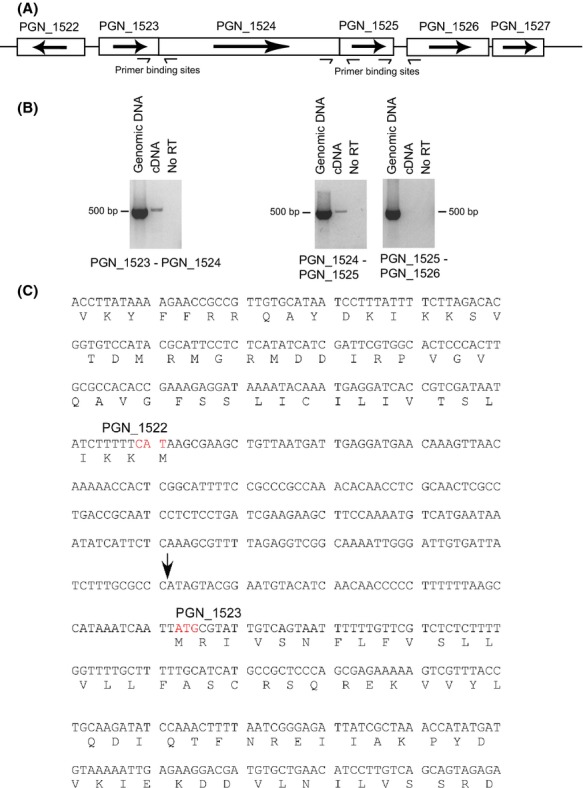
Physical and transcriptional organization of the *ptk1* region in *Porphyromonas gingivalis* 33277. (A) Genetic organization of the *ptk1* region. Transcriptional direction is indicated with arrows. Primer binding sites are indicated for reverse transcriptase PCR (RT-PCR). (B) RT-PCR of genomic or cDNA with primers spanning the intergenic regions between the open reading frames. A negative control without reverse transcriptase was included (No RT). (C) Part of the PGN_1522 and PGN_1523 coding regions with amino acid sequence along with the untranscribed intergenic region. The transcriptional start site for the PGN_1523-1525 codon was mapped by 5′ RACE to an A (indicated with arrow) 52 bp upstream of the methionine ATG start codon (red font) of PGN_1523. The start codon of the upstream PGN_1522, transcribed in the opposite direction, is indicated (red font).

The region of the *P. gingivalis* 33277 genome in which *ptk1* is located contains a cluster of six genes (PGN_1523 – PGN_1528) in the same orientation and in close physical association (Naito et al. 2008). Flanking *ptk1* upstream (6 bp gap) is PGN_1523, annotated as a polysaccharide export gene which shows homology to *wza* in *E. coli*, a gene involved in Group 1 capsule synthesis (Whitfield 2006). Immediately downstream (9 bp gap) of *ptk1* is PGN_1525 annotated as a capsular polysaccharide biosynthesis gene. Further downstream, PGN_1526, PGN_1527, and PGN_1528 are annotated as hypothetical proteins. To determine the operon structure in the *ptk1* region, reverse transcriptase PCR (RT-PCR) was employed to amplify 500 bp fragments spanning each of the respective genes being analyzed (Fig. 2A). A positive control of chromosomal DNA, and a negative control of no RT were included in each experiment. An amplification product was observed in the cDNA template spanning PGN_1523 and *ptk1,* and also between *ptk1* and PGN_1525 (Fig. 2B). In contrast, no amplification product was observed when primers were used to amplify a region between PGN_1525 and the downstream gene PGN_1526, consistent with the end of the operon at PGN_1525. Primers within PGN_1526 successfully amplified cDNA, demonstrating that the negative result was not due to degradation of PGN_1526 mRNA (Fig. S2). A control of primers spanning PGN_1522 (transcribed in the opposite direction) and PGN_1523 did not produce a product from cDNA (not shown). These results establish that PGN_1523, *ptk1,* and PGN_1525 are transcriptionally linked.

To further investigate this operon and the tss, RACE was utilized to identify the tss of PGN_1523. Six independent RACE reactions mapped the tss to an A residue 52 bp upstream of ATG start codon of PGN_1523 (Fig. 2C). The proximity of the tss of PGN_1523 relative to the start codon is similar to that of the fimbrial genes in *P. gingivalis* and closer than reported for protease genes such as *rgpA* and *rgpB* (Jackson et al. 2000; Park et al. 2007). Manual inspection of the sequence revealed a possible P3'' −10 element (Jackson et al. 2000) TATCTT centered −10/11 upstream of the tss.

### Dephosphorylation of Ptk1 by Ltp1

**Figure 3:**
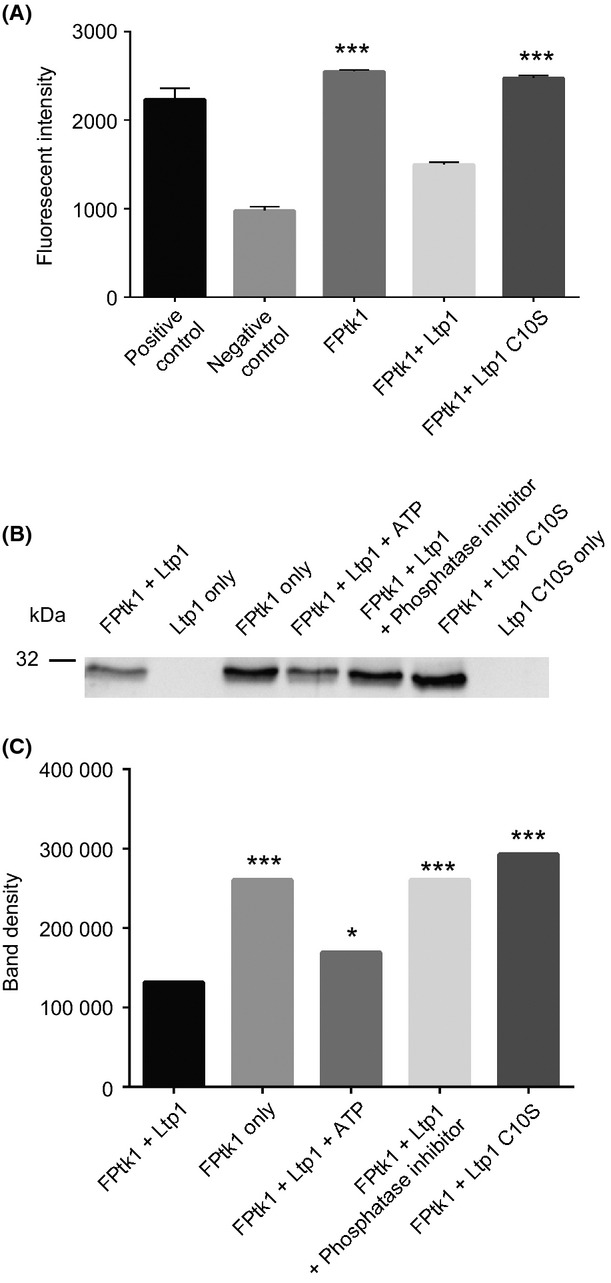
Dephosphorylation of FPtk1 by Ltp1. (A) Incubation of FPtk1 with Ltp1 results in dephosphorylation and reduced kinase activity in the tyrosine kinase assay. Substitution of Cys with Ser at amino acid 10 (Ltp1 C10S) in the catalytically active domain of Ltp1 restored kinase activity. ***P < 0.001 compared to negative control. (B) Western immunoblot of FPtk1 incubated with Ltp1 or Ltp1 C10S, and with ATP or phosphatase inhibitor as indicated. The phosphorylation level of FPtk1 was determined by Western blotting with phosphotyrosine antibodies. (C) Densiometric analysis of band intensity from the blot in (B) using ImageJ to quantitate band density. *P < 0.05; ***P < 0.001 compared to FPtk1 + Ltp1.

In the *P. gingivalis* 33277 genome Ptk1 is the only predicted BY kinase, and Ltp1 is the only predicted tyrosine phosphatase. We hypothesized, therefore, that Ptk1 is the cognate kinase of the Ltp1 tyrosine phosphatase, and the ability of Ltp1 to dephosphorylate Ptk1 was examined. Figure 3A shows that incubation of Ptk1:541-821 with Ltp1 resulted in diminished kinase activity when measured using a fluorescence-based tyrosine kinase assay. Furthermore, reduced tyrosine phosphorylation of Ptk1:541-821 after reaction with Ltp1 was also observed by Western blotting with specific phosphotyrosine antibodies (Fig. 3B and C). Addition of exogenous ATP, to allow autophosphorylation, or a phosphatase inhibitor suppressed the action of Ltp1 and restored the phosphorylated state of Ptk1:541-821 (Fig. 3B and C). The catalytic domain of Ltp1 has been defined, and conversion of the cysteine at position 10 to serine prevents nucleophilic activity and subsequent phosphotransfer (Maeda et al. 2008). When catalytically inactive Ltp1 (C10S) was incubated with Ptk1:541-821, dephosphorylation of the kinase did not occur. Collectively these results establish Ptk1 as one of the substrates for Ltp1 and confirm that the phosphatase activity of Ltp1 is responsible for modification of Ptk1. The extent to which other enzymes can modify Ptk1 and the occurrence of Ltp1-mediated dephosphorylation of Ptk1 in vivo remain to be confirmed.

### Ptk1 is involved in community development with *S. gordonii*

**Figure 4:**
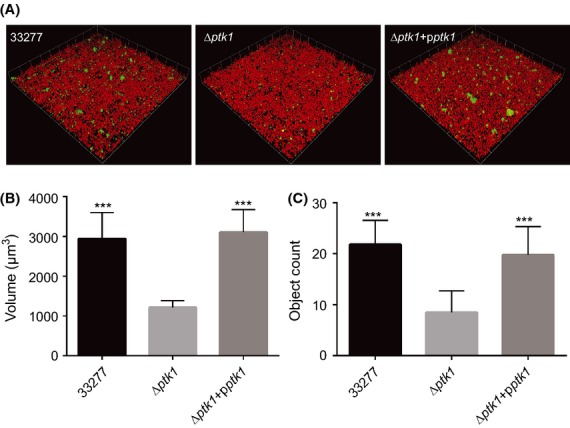
*ptk1* is required for maximal *Porphyromonas gingivalis*–*Streptococcus gordonii* community development. (A) CSLM visualization of *P. gingivalis* 33277, Δ*ptk1,* and Δ*ptk1* + p*ptk1* (green) reacted with a substrate of *S. gordonii* (red) for 18 h. A series of 0.2 *μ*m-deep optical fluorescent *x–y* sections (213 × 213 *μ*m) were collected using an Olympus FV500 confocal microscope, digitally reconstructed into a 3D image and quantitated using Volocity software. (B) Total *P. gingivalis* biovolume was obtained with the 3D Find Objects function of Volocity. (C) Numbers of microcolonies using an object size cut-off algorithm of greater than 30 *μ*m for the total 3D volume for *P. gingivalis* fluorescence. Error bars are SD, *n* = 3. ***P < 0.001 compared to Δ*ptk1*.

Ltp1 is a component of a regulatory pathway involved in interspecies communication between *P. gingivalis* and *S. gordonii*. Binding of the Mfa1 fimbrial adhesin of *P. gingivalis* to the SspA/B streptococcal proteins upregulates Ltp1 and leads to constraint of *P. gingivalis* accumulation (Simionato et al. 2006). Thus, we predicted that Ptk1 would have an opposing effect and promote *P. gingivalis*–*S. gordonii* heterotypic community formation. Consistent with this, the Δ*ptk1* strain of *P. gingivalis* demonstrated a reduced accumulation on a *S. gordonii* substrate compared to the parental strain (Fig. 4A and B). Complementation of Δ*ptk1* with the wild type *ptk1* allele in trans restored the levels of *P. gingivalis* biovolume in the dual species accumulations.

### Ptk1 regulates CdhR and Mfa1 expression

**Figure 5:**
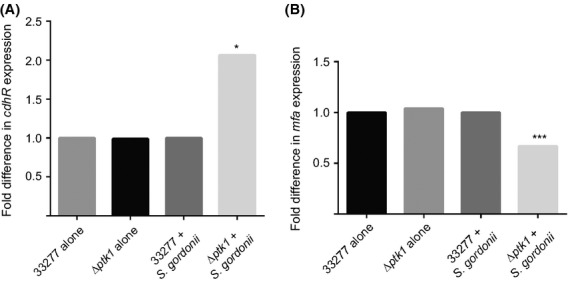
Ptk1 regulates *cdhR* (A) and *mfa1* (B) expression in *P. gingivalis*–*S. gordonii* communities. Messenger RNA extracted from *P. gingivalis* 33277 or Δ*ptk1* cells alone or in a community with *S. gordonii* was analyzed by qRT-PCR. 16s rRNA was used for normalization. *P < 0.05; ***P < 0.001 compared to 33277.

Previous studies have shown that Ltp1 acts indirectly on the transcriptional regulator CdhR, and that loss of Ltp1 prevents a *S. gordonii* induced increase in expression of *cdhR* at the transcriptional level (Chawla et al. 2010). As Ltp1 inactivates Ptk1, we reasoned that Ltp1 is epistatic to Ptk1 in the regulation of CdhR, and hence loss of Ptk1 would increase *cdhR* expression induced by *S. gordonii*. Quantitative RT-PCR showed that *cdhR* mRNA levels were increased in the *ptk1* mutant compared to wild type, in the context of a community with *S. gordonii* (Fig. 5A). Thus, Ptk1 normally serves to reduce expression of *cdhR* and the inactivation of Ptk1 by Ltp1 leads to higher amounts of *cdhR* mRNA. CdhR is a suppressor of transcription of *mfa1,* and the Mfa1 component fimbriae effectuate adhesion with *S. gordonii*. Quantitative RT-PCR also established that *mfa1* mRNA levels were reduced in the Δ*ptk1* mutant in the context of a dual species community, consistent with an increase in CdhR (Fig. 5B).

### Ptk1 controls extracellular polysaccharide

**Figure 6:**
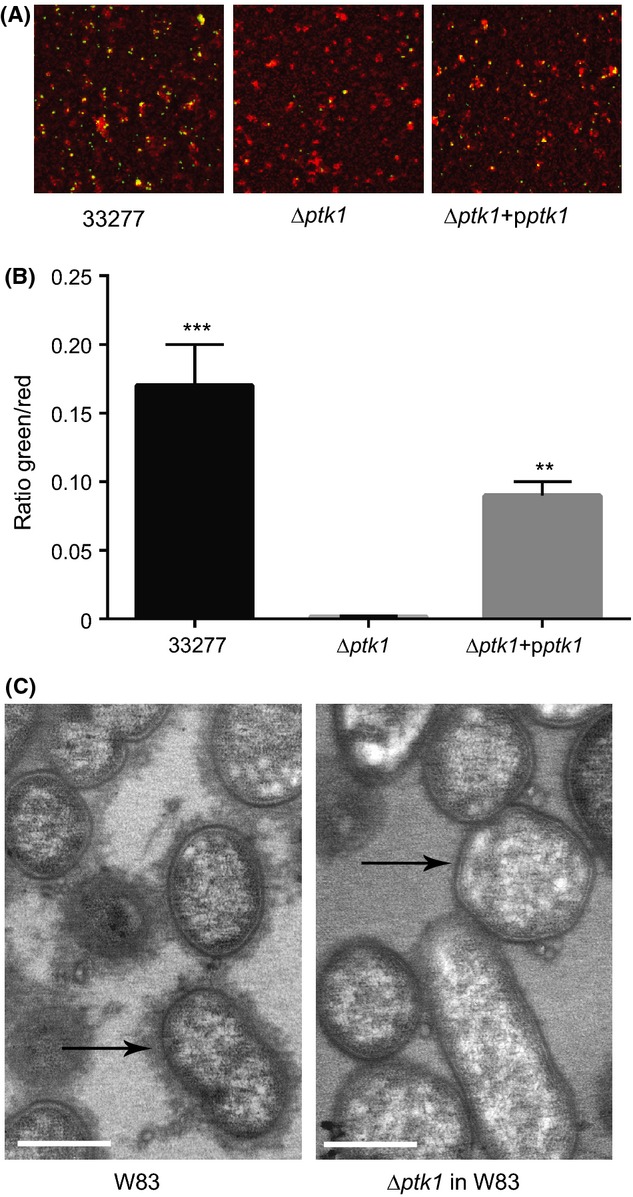
Exopolysaccharide production by *P. gingivalis* is reduced in mutants lacking *ptk1*. (A) Exoploysaccharide produced by *P. gingivalis* 33277 strains was stained with FITC-labeled concanavalin A and wheat germ agglutinin. Bacterial cells were stained with Syto-17. Fluorescent images were collected by confocal microscopy. (B) Ratio of lectin binding (green) to whole cell staining (red) from images in (A) using Volocity software. Error bars are SD, *n* = 3. **P < 0.01; ***P < 0.001 compared to Δ*ptk1* (C) Transmission electron microscopy analysis of *P. gingivalis* W83 wild type and *ptk1* deficient mutant. Ruthenium red staining was employed to visualize capsule. Arrows indicate presence of surface capsule in W83 versus reduced capsule in Δ*ptk1*. Magnification, ×27,500. Scale bar = 0.5 *μ*m.

A major function of the *E. coli* Wzc tyrosine kinase is in the synthesis and export of capsular exopolysaccharide (Whitfield 2006; Grangeasse et al. 2012). Moreover, exopolysaccharide often plays a role in the development of biofilm communities, and Ltp1 was found to impact exopolysaccharide production in *P. gingivalis* (Maeda et al. 2008). To begin to assess the role of Ptk1 in exopolysaccharide production, *P. gingivalis* was stained with fluorescently labeled lectins concanavalin A and wheat germ agglutinin. Exopolysaccharide levels were then determined by confocal microscopy and quantitative image analysis, and normalized to the levels of Syto-17 labeled *P. gingivalis* (Fig. 6A and B). Polysaccharide production was decreased over 10-fold in Δ*ptk1,* and increased in the complemented strain to levels which while less, were statistically no different from the parental strain. Although strain 33277 produces extracellular polysaccharide, it is not organized into a capsule (Maeda et al. 2008), and thus to obtain evidence for the role of Ptk1 in capsule production in *P. gingivalis* we generated the Δ*ptk1* mutation in the encapsulated strain W83. Examination of parental and mutant strains by transmission electron microscopy with ruthenium red staining for polysaccharide (Fig. 6C) showed that mutation of *ptk1* in a W83 background results in a severely diminished in surface capsule. Only small tufts of capsule were visible remaining on the surface of the bacterial cells. A negative control of strain 33277 displayed diffuse and unorganized material staining with ruthenium red (Fig. S3). These data demonstrate that Ptk1 in *P. gingivalis* plays a similar role to homologs in other species regarding capsule synthesis. However, in *P. gingivalis,* which encompasses a number of noncapsulated strains (Aduse-Opoku et al. 2006), tyrosine kinase activity also controls interspecies community formation.

### Substrate phosphorylation by Ptk1

**Figure 7:**
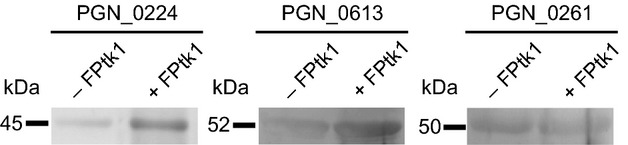
The capsule associated UDP-acetyl-mannosamine dehydrogenase (PGN_0224), and UDP-glucose 6-dehydrogenase (PGN_0613) are phosphorylated by Ptk1. Recombinant protein substrates were incubated with FPtk1 (Ptk1:541-821) in the presence of ATP and a phosphatase inhibitor. PGN_0261 (a sigma-54-dependent transcriptional regulator) was used as a control protein. The phosphorylation level of protein substrates was determined by Western blotting with phosphotyrosine antibodies.

In *E. coli,* the tyrosine kinase Wzc can phosphorylate the enzyme UDP-glucose dehydrogenase (Ugd), which participates in the synthesis of exopolysaccharide (Grangeasse et al. 2003). In capsule production by *Staphylococcus aureus*, the substrate for tyrosine kinase Cap5B_2_ has been defined as Cap5O, a UDP-acetyl-mannosamine dehydrogenase (Gruszczyk et al. 2011). Hence, we tested the ability of Ptk1 to phosphorylate the *P. gingivalis* homologs of Ugd and Cap5O (PGN_0613 and PGN_0224, respectively). Recombinant proteins were reacted with Ptk1:541-821 in the presence of ATP. Probing Western blots with tyrosine phosphorylation specific antibodies showed an increase in tyrosine phosphorylation of both PGN_0224 and PDN_0613 when reacted with Ptk1 (Fig. 7). In contrast, the tyrosine phosphorylation level of a control protein, PGN_0261 a sigma-54-dependent transcriptional regulator (Naito et al. 2008), was not increased by Ptk1. These results imply that in *P. gingivalis* both UDP-acetyl-mannosamine dehydrogenase and UDP-glucose dehydrogenase are substrates for tyrosine kinase activity, and their phosphorylation is likely important for exopolysaccharide production.

## Discussion

While much is known regarding the accumulation of bacteria into monospecies biofilms, less information is available regarding the molecular basis of polymicrobial bacterial community formation and development. Many human diseases such as wound infections, peritonitis, otitis media, and periodontal disease involve communities of bacterial species displaying polymicrobial synergy whereby one organism facilitates the colonization or pathogenicity of another (Ramsey and Whiteley 2009; Ramsey et al. 2011; Peters et al. 2012; Korgaonkar et al. 2013). In periodontal disease, virulence of the keystone periodontal pathogen *P. gingivalis* is increased in the context of a community with *S. gordonii* (Whitmore and Lamont 2011; Hajishengallis and Lamont 2012), and these two organisms are associated in vivo (Slots and Gibbons 1978). *P. gingivalis* and *S. gordonii* interact on a number of levels. Two adhesin-receptor pairs mediate interspecies attachment (Kuboniwa and Lamont 2010) and within the dual species community both *P. gingivalis* and *S. gordonii* differentially express a substantial proportion of their proteomes as they adjust to the altered microenvironment (Kuboniwa et al. 2009). Optimal dual species community development depends on the activity of pathways that can either promote or constrain *P. gingivalis* accumulation. Our previous studies have identified a *P. gingivalis* tyrosine phosphatase, Ltp1, as a component of a signaling pathway that restricts community development (Maeda et al. 2008). Ltp1 functions through increasing the activity of the transcription factor, CdhR, which suppresses expression of the fimbrial adhesin Mfa1 and the LuxS enzyme responsible for AI-2 production (Chawla et al. 2010).

In this study we identify for the first time a BY kinase, Ptk1, in *P. gingivalis* and present evidence that Ptk1 is an important component of the signaling pathways that control *P. gingivalis* synergistic interactions with *S. gordonii*. Loss of Ptk1 activity, through deletion of the *ptk1* gene, decreases the degree of dual species community formation. Hence, Ptk1 is required for optimal levels of *P. gingivalis* accumulation with *S. gordonii*. The phenotype of the *ptk1* mutant is in contrast to that of the *ltp1* mutant which displays elevated *P. gingivalis* accumulation with *S. gordonii* (Maeda et al. 2008), indicating that Ptk1 and Ltp1 comprise a signaling rheostat for community development. Tight control of community development may be necessary for *P. gingivalis* in the dynamic conditions of the oral cavity where changes in oxygen levels and nutrient availability can occur frequently and rapidly. Further evidence that Ptk1 and Ltp1 comprise a cognate kinase-phosphatase pair was provided by the finding that Ltp1 can reversibly dephosphorylate Ptk1. As the activity of BY kinases can be regulated by the degree of autophosphorylation (Doublet et al. 2002; Paiment et al. 2002), the levels and activity of Ltp1 may fine-tune the functionality of Ptk1 through affecting autophosphorylation. A similar model has been established for the control of the *E. coli* BY kinases Wzc and Etk by the Wzb and Etp phosphatases, whereby the phosphatase regulates cycling between highly phosphorylated and reduced phosphorylation states of the cognate kinase (Bechet et al. 2009; Nadler et al. 2012; Temel et al. 2013).

Dual species *P. gingivalis*–*S. gordonii* community development is initiated by the binding of the *P. gingivalis* Mfa1 fimbrial adhesin to the BAR domain of the streptococcal SspA/B protein (Park et al. 2005; Daep et al. 2006). Expression of *mfa1* is under negative control by CdhR, and our data showed that Ptk1 suppresses transcription from the *cdhR* gene and consequently increases *mfa1* mRNA levels. In this manner the action of Ptk1 will favor *P. gingivalis*–*S. gordonii* community development. The extent to which Ptk1 can also directly phosphorylate CdhR is currently under investigation. Mfa1 is a major adhesin and immunogen of *P. gingivalis,* and is positively controlled by the FimS/FimR TCS (Wu et al. 2007; Lo et al. 2010) and negatively regulated by the Clp proteolytic chaperone system components ClpXP (Capestany et al. 2008). Thus, several signaling systems in *P. gingivalis* intersect at *mfa1* transcription and likely provide additional flexibility for *P. gingivalis* to integrate diverse environmental cues into the regulation of community formation.

In many of the bacteria studied to date tyrosine kinases are involved in the production and export of extracellular polysaccharide. A related role was also evident in *P. gingivalis* as loss of Ptk1 causes a reduction in exopolysaccharide production both in strain 33277 in which the exopolysaccharide is not structured as a distinct capsule, and in the capsulated strain W83. In the archetypal *E. coli* exopolysaccharide production system, the Wzc kinase is located in the inner membrane in close association with the lipoprotein Wza which forms a translocation channel through which capsule products are exported (Whitfield 2006). In *P. gingivalis* the Wza homolog PGN_1523 is immediately upstream of Ptk1 and co-transcribed in a three-gene operon. The third gene in the operon is annotated as a capsular polysaccharide biosynthesis protein and contains a CapC domain. It is reasonable to propose, therefore, that Ptk1 plays a similar role to Wzc and that Ptk1 and PGN_1523 interact to allow export of exopolysaccharide components. In support of this, we found that Ptk1 can phosphorylate the *P. gingivalis* homolog of Ugd, a defined substrate for Wzc (Grangeasse et al. 2003). Additionally, in *S. aureus* UDP-acetyl-mannosamine dehydrogenase has been identified as a substrate of the tyrosine kinase Cap5B_2_ (Gruszczyk et al. 2011), and Ptk1 phosphorylated the homologous *P. gingivalis* enzyme, PGN_0224. The role of exopolymeric substances, including polysaccharides, in single-species biofilms is well established. These polymers can form a three-dimensional cohesive network that mechanically stabilizes biofilm structure through interconnectivity among bacterial cells and between cells and the substratum (Flemming and Wingender 2010). Exopolysaccharide production by *P. gingivalis* has also been shown to be important for the formation of dual species communities with *S. gordonii* (Hashino et al. 2013). Thus, Ptk1 may exert control over *P. gingivalis*–*S. gordonii* communities through two distinct pathways involving production of the Mfa1 adhesin and exopolysaccharide.

BY kinases are now recognized as associated with a wide range of bacterial properties including antibiotic resistance, phage lysogenization, stress responses, and production of secondary metabolites (Grangeasse et al. 2007, 2012; Whitmore and Lamont 2012). Furthermore, the addition of a large, negatively charged phosphoryl group to a protein can influence both cellular location and the overall protein interactome (Stulke 2010). As the number of phosphoproteomic databases increase, the full range of BY kinase functions will become more apparent. A role in polymicrobial synergy as established here, defines a novel function for a BY kinase. As bacteria frequently inhabit complex multispecies communities, an important future goal will be to determine the extent to which BY kinases control synergistic interactions in other mixed infections.

## Supporting Information

**Figure S1.** Growth of *Porphyromonas gingivalis* 33277, Δ*ptk1,* and Δ*ptk1 +* p*ptk1* at 37°C in supplemented TSB, monitored by OD_600_. Data are from one representative experiment of three biological replicates.

**Figure S2.** RT-PCR of genomic or cDNA with primers PGN_1526F: GAGGGGTGCTCTTTTTTCGTCG and PGN_1526R: AAGGCTTCCGTCTCGGATCGTG within the PGN_1526 gene. A negative control without reverse transcriptase was included (No RT).

**Figure S3.** Transmission electron microscopy of *Porphyromonas gingivalis* 33277 stained with ruthenium red. Magnification, ×27,500.

**Table S1.** Primers used in this study.
