# Supplementary figures and images for: Characterization of a bacterial tyrosine kinase in Porphyromonas gingivalis involved in polymicrobial synergy
Source: Microbiologyopen. 2014 May 9;3(3):383–94. doi: 10.1002/mbo3.177 (PMC4082711; doi:10.1002/mbo3.177)

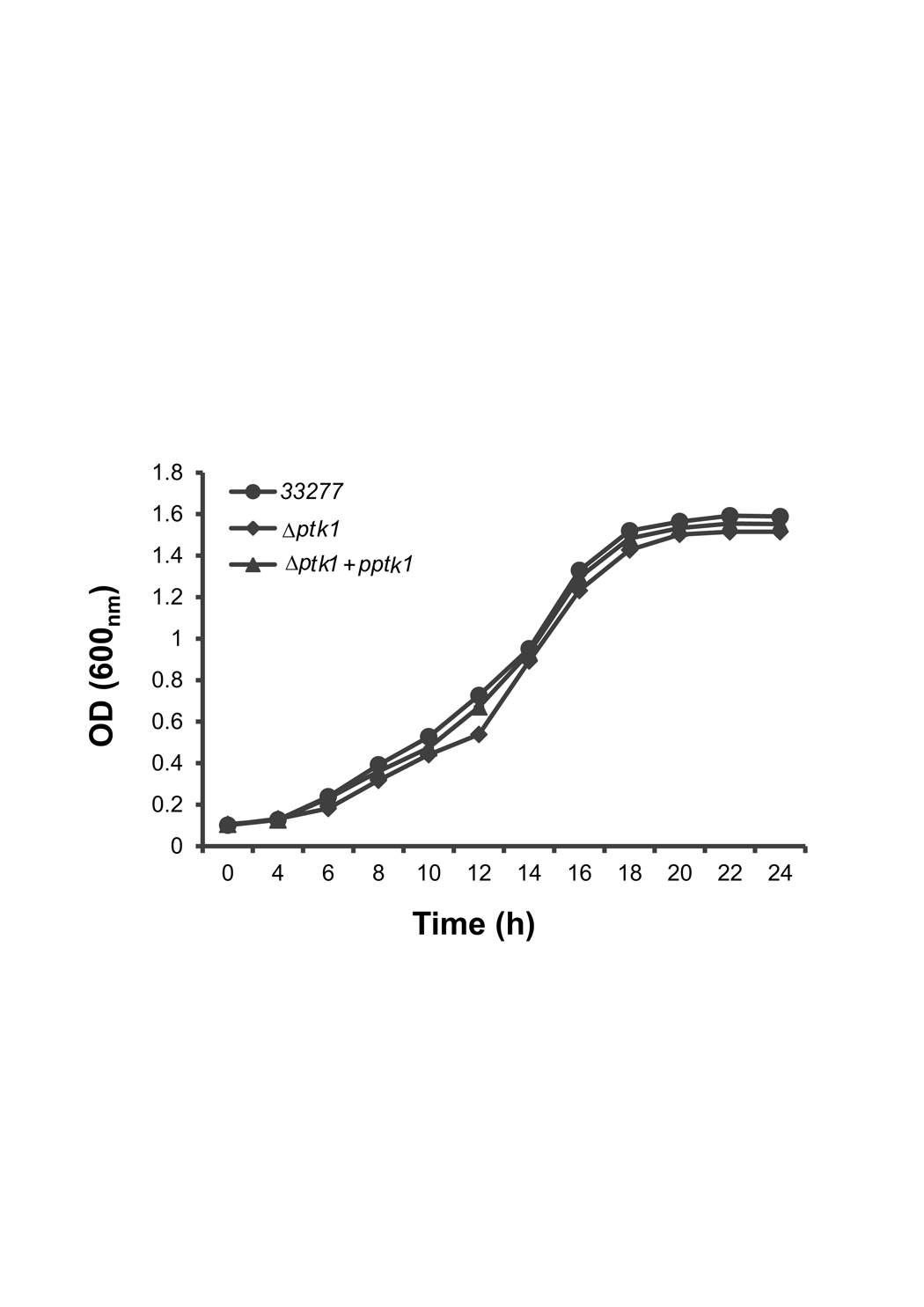

Supplement: Supplementary file 1 — Figure S1. Growth of Porphyromonas gingivalis 33277, Δptk1, and Δptk1 + pptk1 at 37°C in supplemented TSB, monitored by OD600. Data are from one representative experiment of three biological replicates. [file mbo30003-0383-sd1.tif]

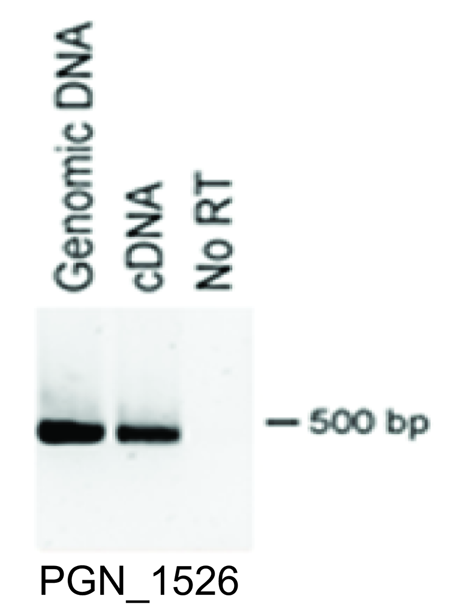

Supplement: Supplementary file 2 — Figure S2. RT-PCR of genomic or cDNA with primers PGN_1526F: GAGGGGTGCTCTTTTTTCGTCG and PGN_1526R: AAGGCTTCCGTCTCGGATCGTG within the PGN_1526 gene. A negative control without reverse transcriptase was included (No RT). [file mbo30003-0383-sd2.tif]

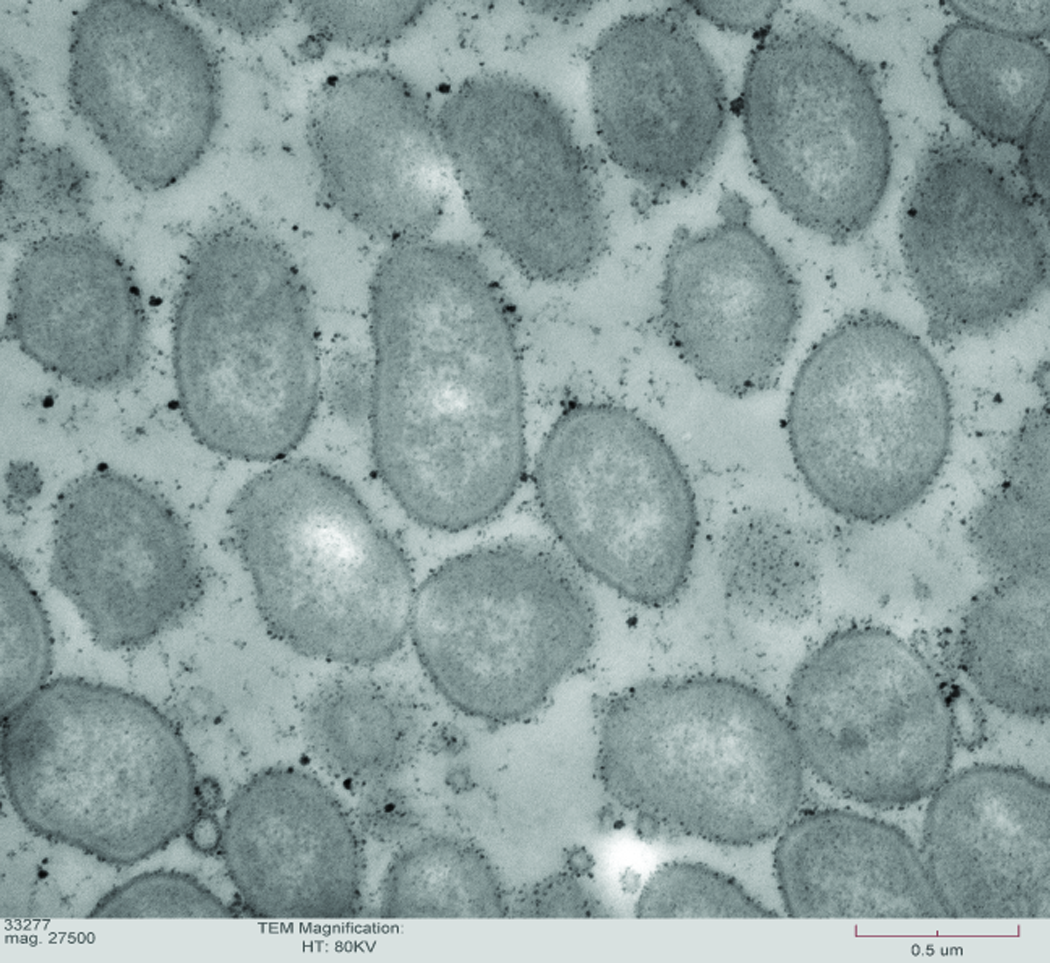

Supplement: Supplementary file 3 — Figure S3. Transmission electron microscopy of Porphyromonas gingivalis 33277 stained with ruthenium red. Magnification, ×27,500. [file mbo30003-0383-sd3.tif]
