# Supplementary material for: Characterization of a bacterial tyrosine kinase in Porphyromonas gingivalis involved in polymicrobial synergy
Source: Microbiologyopen. 2014 May 9;3(3):383–94. doi: 10.1002/mbo3.177 (PMC4082711; doi:10.1002/mbo3.177)
Supplement: Supplementary file 4 — Table S1. Primers used in this study. [file mbo30003-0383-sd4.docx]

|  | Table S1. Primers used in this study |  |
| --- | --- | --- |
| Name | Primer sequence (5’ – 3’) | Function |
| A1524usF | GTATTGTCAGTAATTTTTTG | Generating fragment upstream of PGN_1524 for use with allelic exchange mutation |
| B1524usR | GGAAGCTATCGGGGGTACCTTCAATTAAGTTCATCTTTC |  |
| C1524dsF | TGTCCCTGAAAAATTTCATCCATAGGATGTTTTCTATTTTCAAG | Generating fragment upstream of PGN_1524 for use with allelic exchange mutation |
| D1524dsF | CTGTATTATAAAAACGATCGA |  |
| A436usF | ttgagaaggacgatgtgctg | Generating fragment upstream of PG436 for use with allelic exchange mutation |
| B436usR | GGAAGCTATCGGGGGTACCggccaatacactcgctacaa |  |
| C436dsF | TGTCCCTGAAAAATTTCATCCgccccatgaagaagagctaa | Generating fragment upstream of PG436 for use with allelic exchange mutation |
| D436dsR | attcggaaccagcccttatc |  |
| ERMf | GGTACCCCCGATAGCTTCC | Erythromycin gene for fusion with upstream and downstream products above |
| ERMr | GGATGAAATTTTTCAGGGACA |  |
| ptcow1524F | ATGAACTTAATTGAAGATTCAAAAAACAC | For cloning full length PGN_1524 into pTCOW |
| ptcow1524R | TTAGCTCTTCTTCATGGGGCC |  |
|  |  |  |
| ltp1C10SF | ATGAAGCCACATAAAATC | Generation of recombinant Ltp1 C10S |
| ltp1C10SR | tcagtcgcatgcggat |  |
| pet200224F | atgaaaaaagcttgtttcatggg | Generation of recombinant PGN_0224 |
| pet200224R | ctacttcttgaagacaccgcag |  |
| pet200613F | atgaaaccatataaggcattgaca | Generation of recombinant PGN_0613 |
| pet200613R | ctacagtttgtgatactttttcccttg |  |
| pet200261F | atgagcaccaatatagatgtacaacag | Generation of recombinant PGN_0261 |
| pet200261R | ctattccagtccatactccttgattt |  |

|  | Table S1 continued. Primers used in this study |  |
| --- | --- | --- |
| Name | Primer sequence (5’ – 3’) | Function |
| RTcdhF | AAGACCTTGGAGCCTGAAGAG | Primers used in gene expression analysis |
| RTcdhR | TCATGATGGCATAGATGGTCA |  |
| RTmfaF | tgcggcgaagtcgtaatg |  |
| RTmfaR | atcttcagcactctcacaag |  |
| RT16sF | AGGAACTCCGATTGCGAAGG |  |
| RT16sR | TCGTTTACTGCGTGGACTACC |  |
|  |  |  |
| 5’ RACE  PGN_1523  inner | TTCAGCACATCGTCCTTCTC | Primers used for 5’ RACE |
| 5’ RACE  PGN_1523  outer | GGTCTCTACTGCTGACAAGG |  |
|  |  |  |
| PGN_1523F | GCCCCGTGTACTATCTGCAT | Reverse-transcriptase PCR primers to determine operon structure |
| PGN_1524R | GCTTTCGGCAAGTGTGTTTT |  |
| PGN_1524F | GCTCTGCCAAGAAGACGAAG |  |
| PGN_1525R | GAAAAATGGCGTCATTCGAT |  |
| PGN_1525F | CTATAGCCCGATTGCTGTCC |  |
| PGN_1526R | CGATGATTGCAAAGAGAGCA |  |
